# Supplementary material for: Kinetochore-microtubule attachments are strengthened by Cnn1 stabilization of Stu2
Source: bioRxiv. 2026 Jul 14:2026.07.13.738289. Preprint. [Version 1] doi: 10.64898/2026.07.13.738289 (PMC13405283; doi:10.64898/2026.07.13.738289)
Supplement: Supplement 1 [file media-1.pdf]

Supplementary Materials for

Kinetochore-microtubule attachments are strengthened by Cnn1 stabilization of Stu2

Nairita Maitra *et al.*

*Corresponding author: Sue Biggins, sbiggins@fredhutch.org

This PDF file includes:

Figs. S1 to S7
Tables S1 to S3

Other Supplementary Materials for this manuscript include the following:

Tables S4 to S6

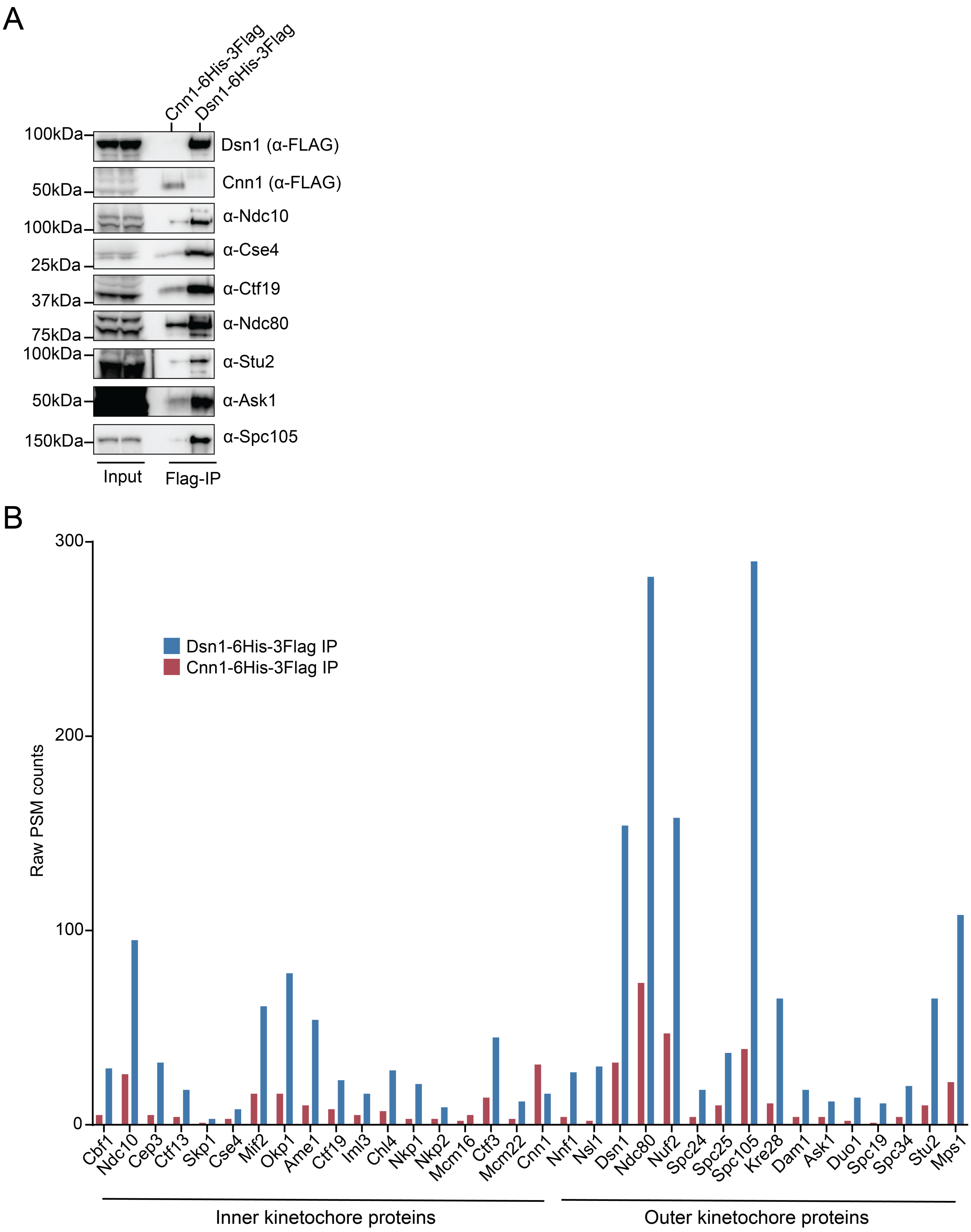

A
Cnn1-6His-3Flag
Dsn1-6His-3Flag
100kDa
Dsn1 (α-FLAG)
50kDa
Cnn1 (α-FLAG)
100kDa
α-Ndc10
25kDa
α-Cse4
37kDa
α-Ctf19
75kDa
α-Ndc80
100kDa
α-Stu2
50kDa
α-Ask1
150kDa
α-Spc105
Input
Flag-IP
B
Dsn1-6His-3Flag IP
Cnn1-6His-3Flag IP
Raw PSM counts
300
200
100
0
Cbf1
Ndc10
Cep3
Ctf13
Skp1
Cse4
Mif2
Okp1
Ame1
Ctf19
Iml3
Chl4
Nkp1
Nkp2
Mcm16
Ctf3
Mcm22
Cnn1
Nnf1
Nsl1
Dsn1
Ndc80
Nuf2
Spc24
Spc25
Spc105
Kre28
Dam1
Ask1
Duo1
Spc19
Spc34
Stu2
Mps1
Inner kinetochore proteins
Outer kinetochore proteins

Fig. S1. Kinetochore proteins that co-purify with Cnn1 and Dsn1.

A. Immunoblot analysis of kinetochore components in Cnn1-Flag or Dsn1-Flag purifications. Cells were arrested in mitosis with benomyl treatment and Cnn1-6His-3Flag (SBY24005) or Dsn1-6His-3Flag (SBY8253) was purified via anti-Flag immunoprecipitation.
B. Mass spectrometry analysis of the kinetochore components from the purifications in (A). Raw peptide spectral match (PSM) counts are presented in the y-axis.

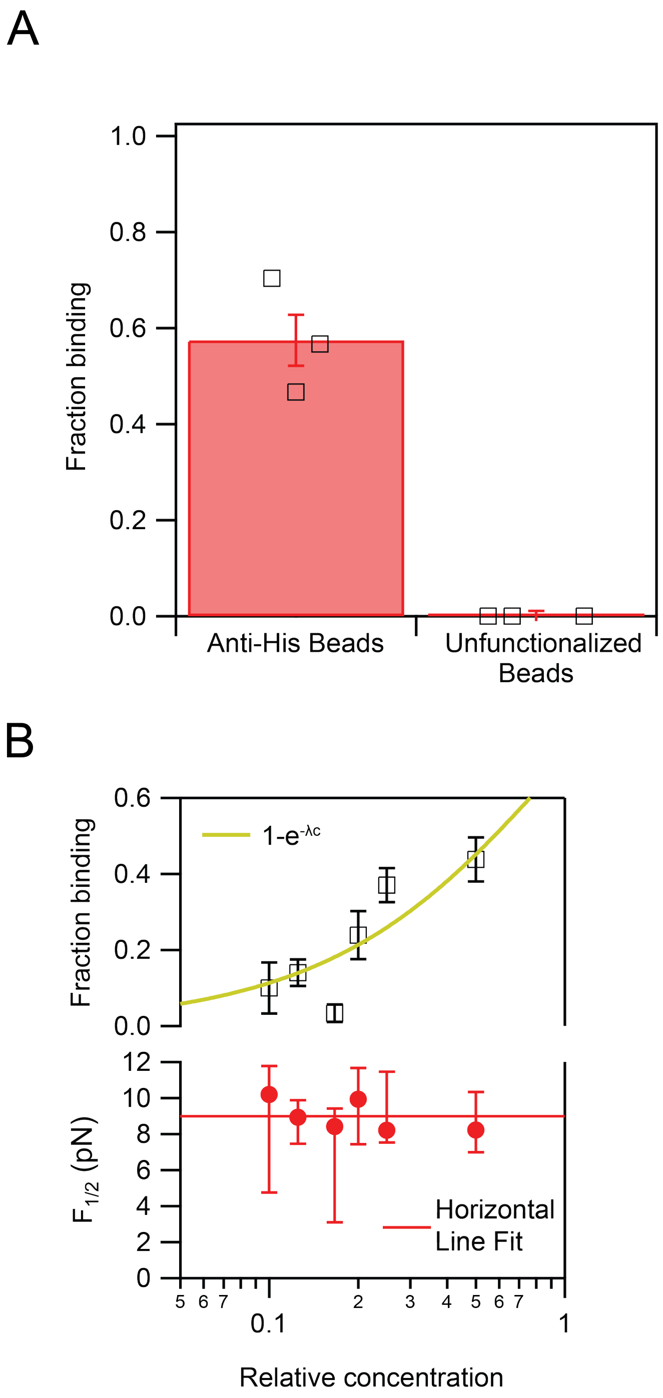

A
Fraction binding
1.0
0.8
0.6
0.4
0.2
0.0
Anti-His Beads
Unfunctionalized Beads
B
Fraction binding
0.6
0.4
0.2
0.0
1-e-λc
F1/2 (pN)
12
10
8
6
4
2
0
Horizontal Line Fit
5
6
7
0.1
2
3
4
5
6
7
1
Relative concentration

Fig. S2. Rupture forces of Cnn1 particles are consistent with single-particle microtubule binding.

A. Fraction of free beads that bound microtubules in the optical trapping assay where Cnn1-6His-3Flag particles were incubated with either anti-His functionalized or unfunctionalized beads. Black squares show binding fractions measured on a single slide.
B. Top: Fraction of Cnn1-decorated beads that bound dynamic microtubules in optical trapping assay following incubation with varying concentrations of Cnn1 particles. The yellow line shows the binding likelihood assuming binding fractions are governed by the Poisson probability that a bead carries an active particle where c is the relative concentration and λ is a fitting parameter. The number of slides used for bead binding per dilution are: c = 0.5: 4 slides, c = 0.25: 6-slides, c = 0.2: 3 slides, c = 0.17: 3 slides; c = 0.125: 5 slides, c = 0.1: 1 slide. Bottom: The 50% survival force ($F_{1/2}$) for dilutions of Cnn1 purified kinetochores on beads. The error bar indicates 95% confidence intervals. The solid red line is a horizontal line fit to $F_{1/2}$. The number of events (n) per dilution was: c = 0.5: n = 117, c = 0.25: n = 96, c = 0.2: n = 58, c = 0.17: n = 14, c = 0.125: n = 82, c = 0.1: n = 11.

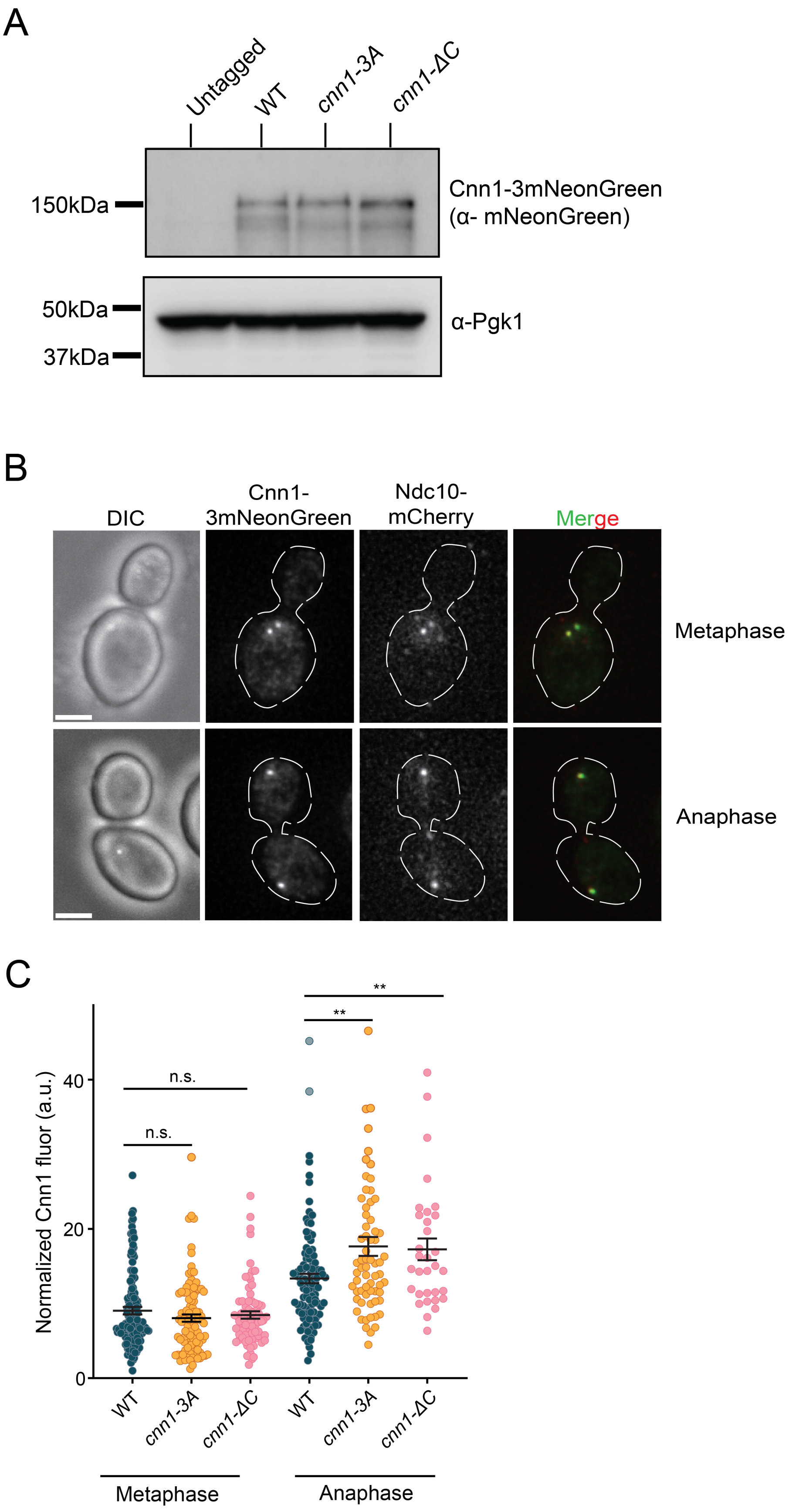

A
Untagged
WT
cnn1-3A
cnn1-ΔC
150kDa
Cnn1-3mNeonGreen
(α- mNeonGreen)
50kDa
37kDa
α-Pgk1
B
DIC
Cnn1-3mNeonGreen
Ndc10-mCherry
Merge
Metaphase
Anaphase
C
Normalized Cnn1 fluor (a.u.)
0
20
40
n.s.
n.s.
**
**
WT
cnn1-3A
cnn1-ΔC
WT
cnn1-3A
cnn1-ΔC
Metaphase
Anaphase

Fig. S3. Mutation of the Cnn1 C-region does not reduce Cnn1 protein levels.

A. Cnn1-3mNeonGreen protein levels were assayed via immunoblot in *CNN1-3mNeonGreen* (SBY24029), *cnn1-3A-3mNeonGreen* (SBY24030), and *cnn1-ΔC-3mNeonGreen* (SBY24031) cells from asynchronously growing cultures. Pgk1 was used as the loading control.
B. Representative images of Cnn1 kinetochore localization during metaphase and anaphase. Cells expressed endogenous Cnn1-3mNeonGreen and endogenous Ndc10-mCherry. Scale bar is 2 μm.
C. Quantification of Cnn1 levels at the kinetochore. *CNN1-3mNeonGreen* (SBY24029), *cnn1-3A-3mNeonGreen* (SBY24030), and *cnn1-ΔC-3mNeonGreen* (SBY24031) cells were released from G1 and harvested at 75 mins and 100 mins to visualize metaphase and anaphase stages, respectively. Cnn1-3mNeonGreen fluor was quantified and normalized to the fiducial marker of Ndc10-mCherry. P-values are calculated by Mann-Whitney test. P-values calculated for metaphase stage, WT vs *cnn1-3A* is 0.21; WT vs *cnn1-ΔC* is 0.85; *cnn1-3A* vs *cnn1-ΔC* is 0.31. For anaphase stage, WT vs *cnn1-3A* is 0.002; WT vs *cnn1-ΔC* is 0.007 and *cnn1-3A* vs *cnn1-ΔC* is 0.942. P-value between WT in metaphase vs WT in anaphase is < 0.0001. Error bars represent S.E.M. n.s. is non-significant. ** represents <0.01.

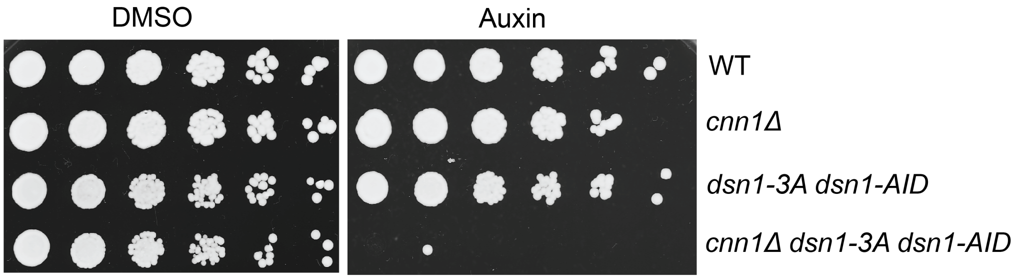

Fig S4. *Cnn1Δ* cells are inviable when the Mis12c pathway is crippled.

Viability of wild type (SBY3), *cnn1Δ* (SBY16332), *dsn1-3A dsn1-AID* (SBY19409), and *cnn1Δ dsn1-3A dsn1-AID* (SBY22387) cells was assayed via serial dilutions (5-fold dilutions) on YPD with either DMSO or 500 μM auxin.

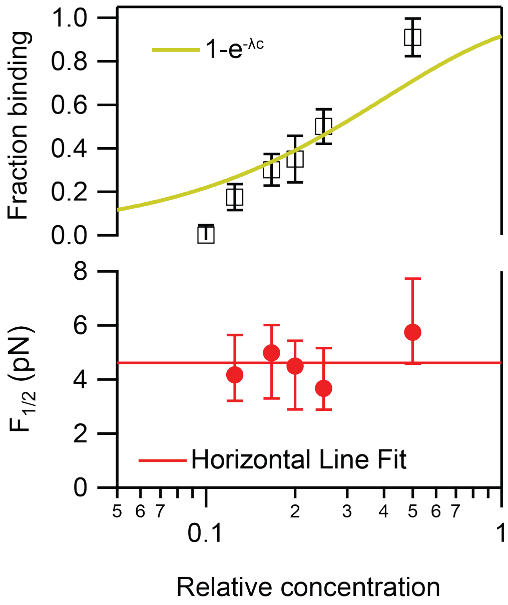

Fig S5. Rupture forces of Cnn1-3A particles are consistent with single-particle microtubule binding.

Top: Fraction of Cnn1-3A particle-decorated beads that bound dynamic microtubules in optical trapping assay following incubation with varying concentrations of Cnn1-3A purifications. The yellow line shows the binding likelihood assuming binding fractions are governed by the Poisson probability that a bead carries an active particle where c is the relative concentration and λ is a fitting parameter. The number of slides used for bead binding at each relative concentration are: c = 0.5: 1 slide, c = 0.25: 2-slides, c = 0.2: 1 slide, c = 0.17: 2 slides, c = 0.125: 2 slides, c = 0.1: 1 slide. Bottom: The 50% survival force ($F_{1/2}$) for dilutions of Cnn1-3A purified kinetochores on beads. The error bar indicates 95% confidence intervals. The solid red line is a horizontal line fit to $F_{1/2}$. The number of events (n) per dilution were: c = 0.5: n = 30, c = 0.25: n = 52, c = 0.2: n = 22, c = 0.17: n = 34, c = 0.125: n = 37, c = 0.1: n = 0.

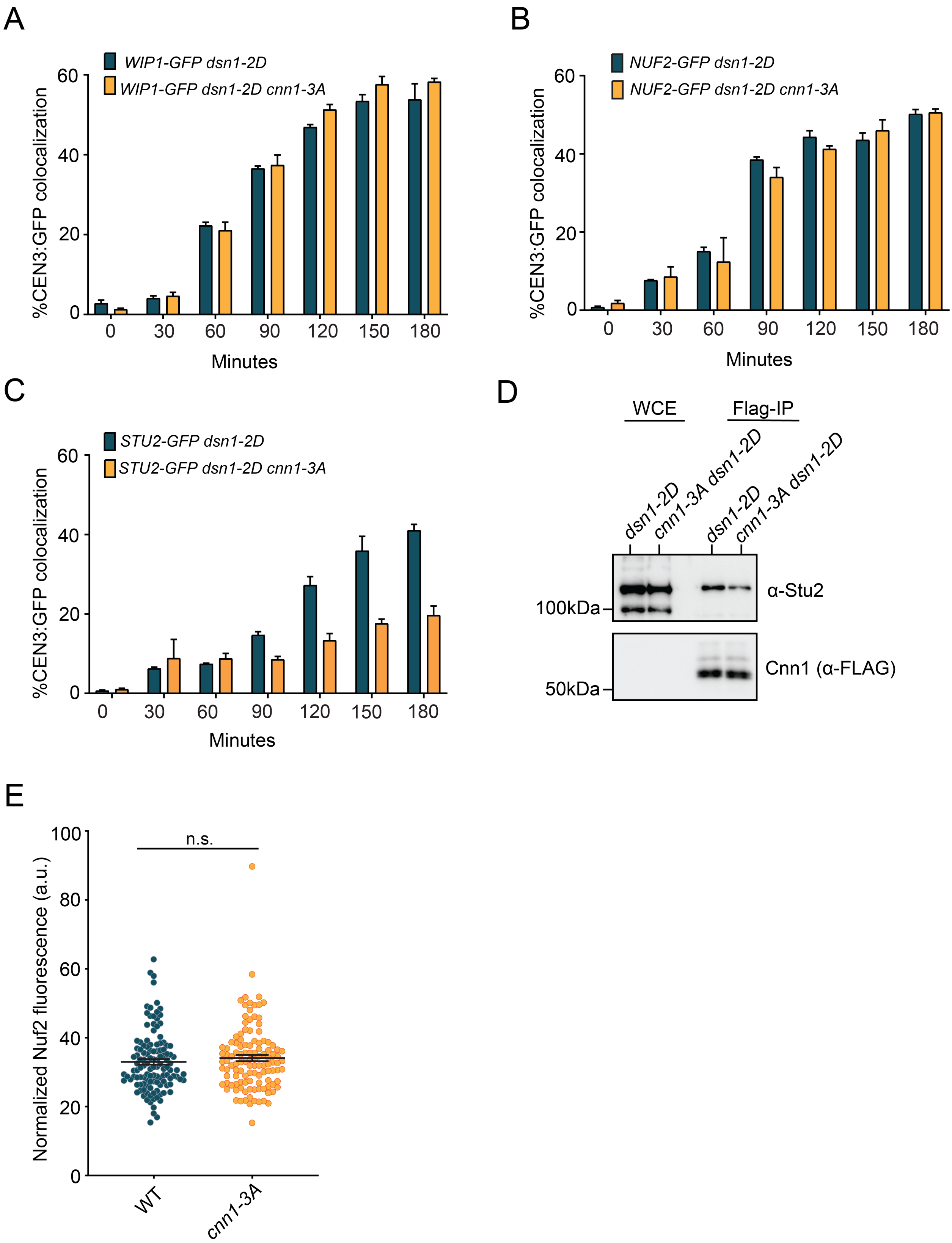
A
WIP1-GFP dsn1-2D
WIP1-GFP dsn1-2D cnn1-3A
%CEN3:GFP colocalization
0
20
40
60
0 30 60 90 120 150 180
Minutes
B
NUF2-GFP dsn1-2D
NUF2-GFP dsn1-2D cnn1-3A
%CEN3:GFP colocalization
0
20
40
60
0 30 60 90 120 150 180
Minutes
C
STU2-GFP dsn1-2D
STU2-GFP dsn1-2D cnn1-3A
%CEN3:GFP colocalization
0
20
40
60
0 30 60 90 120 150 180
Minutes
D
WCE
Flag-IP
dsn1-2D
cnn1-3A dsn1-2D
dsn1-2D
cnn1-3A dsn1-2D
α-Stu2
100kDa
Cnn1 (α-FLAG)
50kDa
E
Normalized Nuf2 fluorescence (a.u.)
n.s.
0
20
40
60
80
100
WT
cnn1-3A

Fig S6. *Cnn1-3A* cells exhibit reduced Stu2 levels at the kinetochore.

A. Percentage of Atto-647 *CEN3* DNA colocalized with Wip1-GFP measured by single molecule TIRF assay using *WIP1-GFP dsn1-2D* (SBY22205) and *WIP1-GFP dsn1-2D cnn1-3A* (SBY24956) lysates. 3000 DNA molecules were imaged at each time point for each strain and biological replicate (n = 3). The time axis represents the incubation time of the DNA with the lysate before it was washed out and TIRFM was performed. P-value calculated from T-test over three experiments at 180 mins is 0.044.
B. Percentage of Atto-647 tagged *CEN3* DNA colocalized with Nuf2-GFP measured by a single molecule TIRF assay using *NUF2-GFP dsn1-2D* (SBY23258) and *NUF2-GFP dsn1-2D cnn1-3A* (SBY24955) cells. 3000 DNA molecules were imaged at each time point for each strain and biological replicate (n = 3). The time axis represents the incubation time of the DNA with the lysate before it was washed out and TIRFM was performed. P-value calculated from T-test over three experiments at 180 mins is 0.8715.
C. Percentage of Atto-647 *CEN3* DNA colocalized with Stu2-GFP measured by single molecule TIRF assay using *STU2-GFP dsn1-2D* (SBY22133) and *STU2-GFP dsn1-2D cnn1-3A* (SBY24954) cells. 3000 DNA molecules were imaged at each time point for each strain and biological replicate (n = 3). The time axis represents the incubation time of the DNA with the lysate before it was washed out and TIRFM was performed. P-value calculated from T-test over three experiments at 180 mins is <0.0001.
D. Cnn1-Flag was immunoprecipitated from benomyl arrested *dsn1-2D* (SBY23855) and *dsn1-2D cnn1-3A* (SBY23856) cells. Stu2 levels were analyzed via immunoblot.
E. Quantification of Nuf2 level at kinetochores in vivo. Wild type cells (SBY24827) and *cnn1-3A* cells (SBY24826) containing Nuf2-GFP and Ame1-mKATE were released from G1 and harvested 75 mins after release to obtain metaphase cells. The fluor intensity of Nuf2-GFP was quantified and normalized to the Ame1-mKate intensity. The p-value (0.323) is calculated from the Mann-Whitney test across 2 experiments (n > 100 cells for each strain). The error bar represents S.E.M. n.s. represents non-significant.

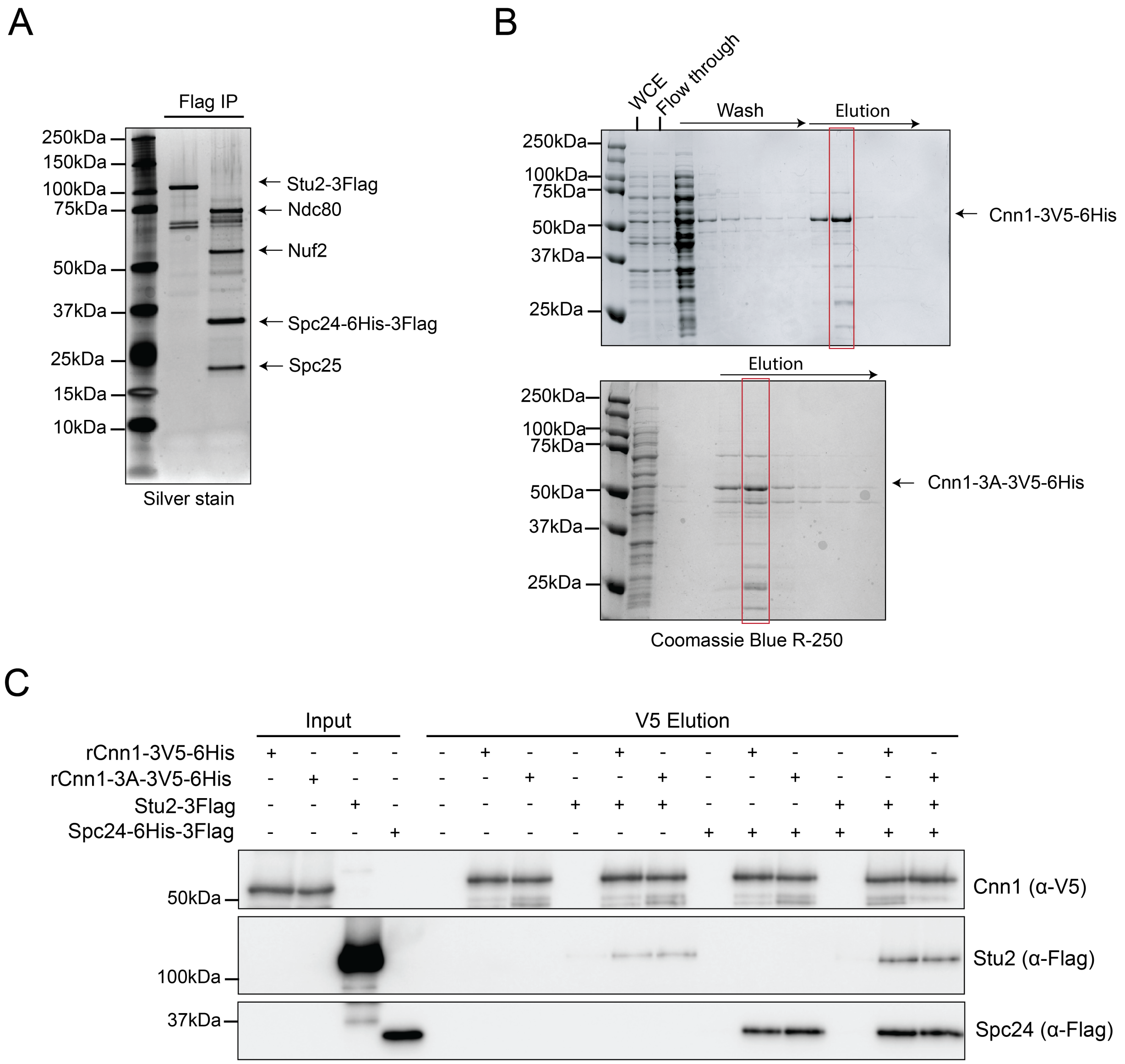

Fig S7. In vitro binding assay between Stu2, Ndc80 and Cnn1.

A. High salt purifications of Stu2-Flag (SBY12275) (left lane) and Ndc80c via Spc24-Flag (SBY14022) (right lane) from budding yeast cells arrested in mitosis by benomyl treatment were analyzed by silver staining of SDS-PAGE.

B. Coomassie staining of SDS-PAGE gels analyzing recombinant wild type Cnn1 (top panel) and Cnn1-3A (bottom panel) proteins expressed in *E. coli* and purified using immobilized metal ion affinity chromatography. Eluate 2, shown in the red box, was used for both Cnn1 and Cnn1-3A binding assays shown in panel C.

C. Binding assays between Cnn1 or Cnn1-3A and Stu2 and Ndc80c. Recombinant Cnn1 and Cnn1-3A also had a 3V5 epitope tag at the C-terminus. Cnn1 was immobilized on V5 beads that

were subsequently incubated with an equal concentration of purified Stu2-Flag, Ndc80c (via Spc24-6His-3Flag) or both proteins for 30 mins at 4 ºC before eluting with V5 peptide (1mg/ml). The proteins were analyzed via immunoblot. The input shows the protein prior to the binding assay.

Supplementary Tables

Table S1: List of strains used in this study

Strain	Genotype
SBY3 (W303)	*MATa ura3-1 leu2-3,112 his3-11 trp1-1 can1-100 ade2-1 bar1-1*
SBY8253	*MATa DSN1-6His-3FLag:URA3*
SBY11586	*MATa CNN1-3Flag:KANMX6*
SBY12275	*MATa STU2-3FLag:KANMX6*
SBY14022	*MATa SPC24-6His-3Flag:URA3 SPC105-3HA-IAA7:KANMX6 trp1-1::pGDP1-OsTIR1:TRP1*
SBY16332	*MATa cnn1Δ::HIS3*
SBY19409	*MATa his3-11::dsn1-3A:HIS3 leu2-3,112::pGDP1-OsTIR1:LEU2 DSN1-3HA-IAA7:KANMx6*
SBY21592	*MATa leu2-3,112::GFP-TUB1:LEU2 MTW1-mCherry:HPH*
SBY21682	*MATa CNN1-3Flag:KANMX6 STU2-3V5:HIS3MX6*
SBY21805	*MATa cnn1-3A-3Flag:KANMX6 STU2-3V5:HIS3MX6*
SBY21908	*MATa leu2-3,112::GFP-TUB1:LEU2 MTW1-mCherry:HPH cnn1-3A*
SBY22078	*MATa cnn1-3A-3FLag:KANMX6*
SBY22133	*MATa STU2-3FLag:KANMX6 dsn1-2D-3Flag:URA3*
SBY22387	*MATa his3-11::dsn1-3A:HIS3 leu2::pGDP1-OsTIR1:LEU2 DSN1-3HA-IAA7:KANMX6 cnn1Δ::HIS3*
SBY22830	*MATa his3-11::dsn1-3A:HIS3 trp1-1:pGDP1-OsTIR1:TRP1 leu2-3,112::GFP-TUB1:LEU2 DSN1-3HA-IAA7:KANMX6 MTW1-mCherry:HPH*
SBY22831	*MATa his3-11::dsn1-3A:HIS3 trp1-1:pGDP1-OsTIR1:TRP1 leu2-3,112::GFP-TUB1:LEU2 DSN1-3HA-IAA7:KANMX6 MTW1-mCherry:HPH cnn1-3A*
SBY22972	*MATa his3-11::dsn1-3A:HIS3 leu2-3,112::pGDP1-OsTIR1:LEU2 DSN1-3HA-IAA7:KANMX6 cnn1-3A-6His-3FLag:URA3*
SBY22977	*MATa his3-11::dsn1-3A:HIS3 leu2-3,112::pGDP1-OsTIR1:LEU2 DSN1-3HA-IAA7:KANMX6 CNN1-6His-3FLag:URA3*
SBY23258	*MATa NUF2-mGFP:KANMX6 dsn1-2D-3Flag:URA3*
SBY24005	*MATa CNN1-6His-3Flag:URA3*
SBY24006	*MATa cnn1-3A-6His-3Flag:URA3*
SBY24029	*MATa NDC10-mCherry:HPH CNN1-3mNeonGreen:HIS3*
SBY24030	*MATa NDC10-mCherry:HPH cnn1-3A-3mNeonGreen:HIS3*
SBY24031	*MATa NDC10-mCherry:HPH cnn1-ΔC-3mNeonGreen:HIS3*
SBY22205	*MATα WIP1-GFP:KANMX6 dsn1-2D-3Flag:URA3*
SBY24336	*MATa ura3-1::pCyc1-GFP12-LacI12:URA3 his3-11::dsn1-3A:HIS3 chr8::CEN256LacO:TRP1 leu2-3,112::pGDP1-OsTIR1:LEU2 DSN1-3HA-IAA7:KANMX6 mad3Δ::NATMX6*
SBY24387	*MATa PDS1-13Myc:LEU2 CNN1-6His-3Flag:URA3*

SBY24389	*MATa his3-11::dsn1-3A:HIS3 leu2-3,112::pGDP1-OsTIR1:LEU2 DSN1-3HA-IAA7:KANMX6 cnn1-3A-6His-3Flag:URA3 mad3Δ::NATMX6*
SBY24391	*MATa mad3Δ::NATMX6*
SBY24542	*MATa ura3-1::pCyc1-GFP12-LacI12:URA3 chr8::CEN256LacO:TRP1 Cnn1-6His-3FLag:URA3 mad3Δ::NATMX6*
SBY24544	*MATa ura3-1::pCyc1-GFP12-LacI12:URA3 chr8::CEN256LacO:TRP1 cnn1-3A-6His-3FLag:URA3 mad3Δ::NATMX6*
SBY24549	*MATa PDS1-13Myc:LEU2 his3-11::dsn1-3A:HIS3 leu2-3,112::pGDP1-OsTIR1:LEU2 DSN1-3HA-IAA7:KANMX6 cnn1-3A-6His-3Flag:URA3 mad3Δ::NATMX6*
SBY24552	*MATa cnn1-ΔC-6His-3Flag:URA3*
SBY24565	*MATa ura3-1::pCyc1-GFP12-LacI12:URA3 his3-11::dsn1-3A:HIS3 chr8::CEN256LacO:TRP1 leu2-3,112::pGDP1-OsTIR1:LEU2 DSN1-3HA-IAA7:KANMX6 cnn1-3A-6His-3Flag:URA3 mad3Δ::NATMX6*
SBY24652	*MATa his3-11::dsn1-3A:HIS3 leu2-3,112::pGDP1-OsTIR1:LEU2 DSN1-3HA-IAA7:KANMX6 cnn1-ΔC-6His-3Flag:URA3*
SBY24738	*MATa PDS1-13Myc:LEU2 CNN1-6His-3Flag:URA3 mad3Δ::NATMX6*
SBY24741	*MATa STU2-GFP:KANMX6 cnn1-3A-6His-3Flag:URA3 AME1-mKATE2:HisMX6*
SBY24782	*MATa STU2-GFP:KANMX6 CNN1-6His-3Flag:URA3 AME1-mKATE2:HIS3MX6*
SBY24789	*MATa PDS-13Myc:LEU2 his3-11::dsn1-3A:HIS3 leu2-3,112::pGDP1-OsTIR1:LEU2 DSN1-3HA-IAA7:KANMX6 cnn1-3A-6His-3Flag:URA3 mad3Δ::NATMX6*
SBY24826	*MATa NUF2-mGFP:KANMX6 cnn1-3A-6HIS-3FLAG:URA AME1-mKate2:HIS3MX6*
SBY24827	*MATa NUF2-mGFP:KANMX6 CNN1-6HIS-3FLAG:URA AME1-mKate2:HISMX6*
SBY24828	*MATa TOR1-1 fprΔ::NATMX6 STU2-FRB:HIS3 NUF2-2FKBP12:TRP1 CNN1-6His-3Flag:URA3*
SBY24829	*MATa TOR1-1 fprΔ::NATMX6 STU2-FRB:HIS3 CNN1-6His-3Flag:URA3*
SBY24830	*MATa TOR1-1 fprΔ::NATMX6 STU2-FRB:HIS3 NUF2-2FKBP12:TRP1 cnn1-3A-6His-3Flag:URA3*
SBY24831	*MATa TOR1-1 fprΔ::NATMX6 STU2-FRB:HIS3 cnn1-3A-6His-3Flag:URA3*
SBY24833	*MATa TOR1-1 fprΔ::NATMX6 STU2-FRB:HIS3 NUF2-2FKBP12:TRP1 his3-11::dsn1-3A:HIS3 leu2-3,112::pGDP1-OsTIR1:LEU2 DSN1-3HA-IAA7:KANMX6 CNN1-6His-3Flag:URA3*
SBY24835	*MATa TOR1-1 fprΔ::NATMX6 STU2-FRB:HIS3 NUF2-2FKBP12:TRP1 his3-11::dsn1-3A:HIS3 leu2-3,112::pGDP1-OsTIR1:LEU2 DSN1-3HA-IAA7:KANMX6 cnn1-3A6His-3Flag:URA3*

SBY24900	*MATa TOR1-1 fprΔ::NATMX6 STU2-FRB:HIS3 his3-11::dsn1-3A:HIS3 leu2-3,112::pGDP1-OsTIR1:LEU2 DSN1-3HA-IAA7:KANMX6 CNN1-6His-3Flag:URA3*
SBY24902	*MATa TOR1-1 fprΔ::NATMX6 STU2-FRB:HIS3 his3-11::dsn1-3A:HIS3 leu2-3,112::pGDP1-OsTIR1:LEU2 DSN1-3HA-IAA7:KANMX6 cnn1-3A-6His-3Flag:URA3*
SBY24954	*MATα STU2-GFP:KANMX6 dsn1-2D-3Flag:URA3 cnn1-3A*
SBY24955	*MATα NUF2-mGFP:KANMX6 dsn1-2D-3Flag:URA3 cnn1-3A*
SBY24956	*MATα WIP1-GFP:KANMX6 dsn1-2D-3Flag:URA3 cnn1-3A*

Table S2: List of plasmids used in this study

Plasmid #	Purpose
pSB1108	To integrate *dsn1-3A* at the ectopic locus of *HIS3*. The plasmid was described in (*1*)
pSB1590	To epitope tag *CNN1* with 6His-3Flag at its C-terminus. The plasmid was described in (*2*).
pSB3405	URA-CEN-sgRNA-Cas9 plasmid targeting ATACAAGATGTTCCCACGAC on *CNN1* to create *cnn1-3A* mutation by CRISPR.
pSB3524	Used as a template to create plasmids pSB3667 and pSB3740. The plasmid was described in (*3*).
pSB3575	To epitope tag *CNN1* with 3mNeonGreen at the C-terminus. The plasmid was described in (*3*).
pSB3667	To induce expression of recombinant Cnn1-3V5-6His in bacteria by IPTG.
pSB3740	To induce expression of recombinant Cnn1-3A-3V5-6His in bacteria by IPTG.
pSB3741	URA-CEN-sgRNA-Cas9 plasmid targeting ATAATAATACAAGATGTTCC on *CNN1* to create *cnn1-ΔC* by CRISPR.

Table S3: List of primers used in this study (5' to 3')

Oligos	Purpose	Sequence
SB660	Reverse primer to sequence *HIS3* locus to verify *dsn1-3A* integration	GTTTAAGGCTAGTGAGTGCG
SB4007	Forward primer to amplify *CNN1* (starting at 184bp) to confirm *cnn1-3A* or *cnn1-ΔC* mutations	GATCCGAACGAAGTACGAAG
SB4126	Reverse primer (~300bp downstream of STOP codon of *CNN1*) to check the 6His-3Flag integration at C-terminus of *CNN1*	ACACTCGTGGCAATGACTCAG
SB4265	Reverse primer to amplify *CNN1* (250bp upstream of the stop codon of *CNN1*) to confirm *cnn1-3A* or *cnn1-ΔC* mutations	AGATCCATTATTTGCAGCGG
SB5178	Forward primer (~300bp upstream of STOP codon of *CNN1*) to check the 6His-3Flag integration at C-terminus of *CNN1*	CCGTTTAAATGTCCCTGCGG
SB7632	Forward primer to clone *CNN1* sgRNA into Cas9 plasmid to create *cnn1-3A*	ggctgggcaacaccttcgggtggcgaatggATACAAGATGTTCCCACGAC
SB7633	Reverse primer to clone *CNN1* sgRNA into Cas9 plasmid to create *cnn1-3A*	attttaacttgctatttctagctctaaaacGTCGTGGGAACATCTTGTAT
SB7634	Repair template to create *cnn1-3A* mutation by CRISPR	TGGCAATGAAACGCAACCTGATTATACTTCCTTATCGCAAACAGTATTTGCAAAATTGCAGGAAAGAGACAAAGGCTTGAAAAGTAGGAAGATTGACCCGgcagcagcaCAAGATGTTCCCACGACAGGACACGAGGATGAATTGACAGTGCACTCTCCAGATAAAGCTAACAGTATCTCTATGGAAGTTTTGCGCACGT

SB8093	Forward primer to sequence *HIS3* locus to verify *dsn1-3A* integration	GATGTTCCCTCCACCAAAG
SB8711	Forward primer to clone *CNN1* sgRNA into Cas9 plasmid to create *cnn1-ΔC*	ggctgggcaacaccttcgggtggcgaatggATAATAATACAAGATGTTCC
SB8712	Reverse primer to clone *CNN1* sgRNA into Cas9 plasmid to create *cnn1-ΔC*	attttaacttgctatttctagctctaaaacGGAACATCTTGTATTATTAT
SB8713	Repair template to create *cnn1-ΔC* mutation by CRISPR	CAACATTGGCAATGAAACGCAACCTGATTATACTTCCTTATCGCAAACAGTATTTGCAAAATTGCAGGAAAGAGACAAAGGCTTGAAAAGTAGGAAGATTGTTCCCACGACAGGACACGAGGATGAATTGACAGTGCACTCTCCAGATAAAGCTAACAGTATCTCTATGGAAGTTTTGCGCACGTCGCCAAGCATTGGAA
SB8800	Reverse primer to epitope tag *CNN1* with 3-mNeonGreen at its C-terminus	TTTTACTCTTTTCTGTACATAAATTGCCACTATTTAATTATTTTCTCTACGGTATCTTTTggcgttagtatcgaatcgacagc
SB8801	Forward primer to epitope tag *CNN1* with 3-mNeonGreen at its C-terminus	CAACATTGGCAATGAAACGCAACCTGATTATACTTCCTTATCGCAAACAGTATTTGCAAAATTGCAGGAAAGAGACAAAGGCTTGAAAAGTAGGAAGATTGTTCCCACGACAGGACACGAGGATGAATTGACAGTGCACTCTCCAGATAAAGCTAACAGTATCTCTATGGAAGTTTTGCGCACGTCGCCAAGCATTGGAA
SB8883	Forward primer to amplify vector pSB3524	GAATTGAAAGTTATTTGTTCcggatccccgggttaattaacatct
SB8884	Reverse primer to amplify vector pSB3524	GCCTTCCTGGGAGTGCTCATTGTATATCTCCTT

		CTTAAAGTTAAACaaaatt atttctagaggg
SB8885	Forward primer to amplify *CNN1* from gDNA	CTTTAAGAAGGAGATA TACAATGAGCACTCCC AGGAAGG
SB8886	Reverse primer to amplify *CNN1* from gDNA	ttaattaacccggggatccgGAAC AAATAACTTTCAATTCT TGATTGAAGTTCTAAG GG
SB9069	Forward primer to epitope tag *CNN1* with 6His-3Flag:URA	GAGCTCGCGCACGAAT TGCTTCCCTTAGAACTT CAATCAAGAATTGAAA GTTATTTGTTCgAATTCa agcttgggttaattaac
SB9070	Reverse primer to epitope tag *CNN1* with 6His-3Flag:URA	TTTTACTCTTTTCTGTA CATAAATTGCCACTATT TAATTATTTTCTCTACG GTATCTTTTgattcggtaatctc cgaacag

Other supplementary materials:

Table S4: Rupture force events and Kaplan Meier curves – provided in excel file

Table S5: Force clamp data and offsets – provided in an excel file

Table S6: Binding rate data – provided in an excel file

References for the supplementary materials

1. B. Akiyoshi *et al.*, The Mub1/Ubr2 ubiquitin ligase complex regulates the conserved Dsn1 kinetochore protein. *PLoS Genetics* **9**, e1003216 (2013).
2. B. Akiyoshi *et al.*, Tension directly stabilizes reconstituted kinetochore-microtubule attachments. *Nature* **468**, 576-579 (2010).
3. D. R. Mallett, M. Jiang, G. M. Minnuto, S. Biggins, Kinetochore clustering is mediated by Mps1 phosphorylation of conserved MELT motifs in Stu1. *bioRxiv*, 2026.2001.2023.701336 (2026).
